# Gelatin/Polycaprolactone Electrospun Nanofibrous Membranes: The Effect of Composition and Physicochemical Properties on Postoperative Cardiac Adhesion

**DOI:** 10.3389/fbioe.2021.792893

**Published:** 2021-12-06

**Authors:** Xingang Wang, Li Xiang, Yongxuan Peng, Zihao Dai, Yuqing Hu, Xiaoting Pan, Xingliang Zhou, Hao Zhang, Bei Feng

**Affiliations:** ^1^ Shanghai Institute of Pediatric Congenital Heart Disease, Shanghai Children’s Medical Center, School of Medicine, Shanghai Jiao Tong University, Shanghai, China; ^2^ Children’s Heart Center, Institute of Cardiovascular Development and Translational Medicine, The Second Affiliated Hospital and Yuying Children’s Hospital, Wenzhou Medical University, Wenzhou, China; ^3^ Department of Pediatric Cardiology, Xinhua Hospital, Shanghai Jiao Tong University School of Medicine, Shanghai, China

**Keywords:** electrospinning, gelatin, polycaprolactone, postoperative adhesion, cardiac surgery

## Abstract

Cardiovascular diseases have become a major threat to human health. The adhesion formation is an inevitable pathophysiological event after cardiac surgery. We have previously shown that gelatin/polycaprolactone (GT/PCL, mass ratio 50:50) electrospun nanofibrous membranes have high potential in preventing postoperative cardiac adhesion, but the effect of GT:PCL composition on anti-adhesion efficacy was not investigated. Herein, nanofibrous membranes with different GT:PCL mass ratios of 0:100, 30:70, 50:50, and 70:30 were prepared via electrospinning. The 70:30 membrane failed to prevent postoperative cardiac adhesion, overly high GT contents significantly deteriorated the mechanical properties, which complicated the suturing during surgery and hardly maintained the structural integrity after implantation. Unexpectedly, the 0:100 membrane (no gelatin contained) could not effectively prevent either, since its large pore size allowed the penetration of numerous inflammatory cells to elicit a severe inflammatory response. Only the GT:PCL 50:50 membrane exhibited excellent mechanical properties, good biocompatibility and effective anti-cell penetration ability, which could serve as a physical barrier to prevent postoperative cardiac adhesion and might be suitable for other biomedical applications such as wound healing, guided tissue or bone regeneration.

## Highlights

• The 70:30 membrane (high gelatin contained) failed to prevent postoperative cardiac adhesion in view of its poor mechanical properties.
• The 0:100 membrane (no gelatin contained) was not suitable to prevent postoperative adhesion either because of its large pore size.
• Only the 50:50 membrane effectively prevented postoperative adhesion and showed great potential in wound healing, guided tissue or bone regeneration.

## 1 Introduction

Cardiovascular diseases have become a major threat to human health, accounting for nearly 31% of deaths worldwide (Leong et al., 2017). The rapid development of interventional therapy and novel drugs has helped a lot to these diseases; nevertheless, surgical treatments still remain essential to many complex heart diseases. Thus, the formation of adhesions between the chest wall and epicardium is an inevitable postoperative pathophysiological event after cardiac surgery. Adhesions caused by the initial surgery make the original anatomical structure disappear or become much more complicated, which increases the risks during reoperations (Shen and Xu, 2014). The prevention of postoperative cardiac adhesion can greatly reduce the reoperative difficulties, intraoperative and postoperative bleeding, surgery time and costs, and even mortality (Lassaletta et al., 2012).

Among the strategies adopted to prevent postoperative cardiac adhesions (e.g., pharmacological intervention (Lopes et al., 2009) or physical barriers (de Oliveira et al., 2014)), using biomaterial-based membranes is believed to be the most promising one. Expanded polytetrafluoroethylene (ePTFE) is possibly the most extensively utilized and evaluated membrane used to prevent postoperative cardiac adhesion (Minale et al., 1988; Jacobs et al., 1996), but it has not been routinely applied in clinic due to certain shortages such as non-degradability, chronic foreign-body reactions, and predispose to infection over time (Ozeren et al., 2002; Tsukihara et al., 2006). Furthermore, several reports have shown that ePTFE membranes may cause severe adhesions and prominent inflammatory reactions as well (Naito et al., 2008; Kaushal et al., 2011).

Electrospinning has become a widely applied method to fabricate nanofibers due to its cost-effectiveness, simplicity, and suitability for mass production (Xue et al., 2019; Keshvardoostchokami et al., 2020; Xu et al., 2021). Owing to its dense fibrous structure, tiny pore size, controllable mechanical properties, and adjustable degradation rate, the electrospun nanofibrous membrane has shown great potential in preventing postoperative adhesions as a physical barrier (Chen et al., 2019; Alimohammadi et al., 2020; Mao et al., 2021). In our pilot study, we have discovered the elelctrospun GT/PCL (mass ratio 50:50) nanofibrous membrane might become a promising barrier to prevent postoperative cardiac adhesion (Feng et al., 2019). However, the GT:PCL composition could affect the mechanical properties, physical structure, biocompatibility, and degradation rate of membranes, and therefore might eventually influence their anti-adhesion efficacy. For instance, Ghasemi et al. reported that compared with GT:PCL 0:100 and 50:50, 30:70 nanofibrous membranes exhibited the most balanced properties to meet all required specifications for nerve regeneration (Ghasemi-Mobarakeh et al., 2008). While Zheng et al. demonstrated that the high PCL content in GT/PCL membranes was unfavorable for 3-D cartilage regeneration and 70:30 might be a relatively suitable ratio (Zheng et al., 2014).

Herein, we fabricated four nanofibrous membranes with different GT:PCL mass ratios of 0:100, 30:70, 50:50, and 70:30 via electrospinning, and comprehensively evaluated their physicochemical properties, biocompatibility, heart/liver/kidney functions, CRP/immunoglobulin levels, and anti-adhesion performance to determine the most optimal composition and provide a strong theoretical basis for the clinical development and applications of these related membranes. Pure GT membrane was not considered since it failed to maintain the structural integrity owing to its poor mechanical properties (Nagarajan et al., 2017).

## 2 Materials and Methods

### 2.1 Materials

GT type A (V900863, 300 Bloom from porcine skin), PCL (440744, average *M*_w_ = 80,000 Da), acetic acid (HAc; 338826, ≥99.8%), and 2, 2, 2-trifluoroethanol (TFE; T63002, ≥99.0%) were purchased from Sigma Aldrich (United States) and were used as received without further purification. Dulbecco’s modified Eagle medium nutrient mix F12 (DMEM/F12, 11330033), fetal bovine serum (FBS; 10099-141C), penicillin-streptomycin (15140-122), and trypsin (25200-056) were purchased from Thermo Fisher Scientific (United States). Anti-cardiac troponin T (ab45932) and anti-vimentin (ab92547) primary antibodies were purchased from Abcam (United Kingdom). Anti-rabbit IgG fluorescent secondary antibody (4412S) was purchased from Cell Signaling Technology (United States). 4′, 6-Diamidino-2-phenylindole (DAPI; C1002) was purchased from Beyotime Biotechnology (China). The neonatal rat/mouse cardiomyocyte isolation kit (nc-6031) was purchased from Cellutron Life Technologies (United States). Live/dead cell viability assay kits (L3224) were purchased from Thermo Fisher Scientific (United States). Cell Counting Kit-8 (CCK-8; CK04) was purchased from Dojindo Laboratories (Kumamoto, Japan). A modified hematoxylin-eosin (H-E) staining kit (G1121) was purchased from Solarbio Technology (China).

### 2.2 Membrane Preparation

GT and PCL with mass ratios of 30:70, 50:50, and 70:30 were dissolved in TFE to produce a total concentration of 10% (w/v), and the solution was supplemented with a small amount of HAc (0.2 vol%) to obtain a transparent GT/PCL solution. In the case of pure-PCL membrane, PCL was dissolved in TFE at a concentration of 12% (w/v) without HAc. After 48-h stirring, the solutions were filled into a 10-ml syringe with a blunt needle (20G) and fed into the electrospinning device at 2 ml/h using a syringe pump (KDS100, KD Scientific, United States). A rotating stainless-steel drum (diameter = 5 cm, rotation speed = 100 rpm) was used as a collector to prepare membranes with uniform thickness. A high voltage (TXR1020N30-30, Teslaman, China) was applied between the needle tip and grounded collector, with the distance between tip and collector maintained at 13 cm. Other detailed electrospinning parameters in fabrication of different GT/PCL nanofibrous membranes were listed in Table 1. Aluminum foil was wrapped on the collector before electrospinning to facilitate membrane collection and tailoring. The obtained membranes were placed in a vacuum oven for at least 1 week to remove the residual solvent for subsequent use.

**TABLE 1:** Parameters used for electrospinning nanofibrous membranes of GT/PCL.

Membranes (GT:PCL)	Concentration (%)	Applied voltage (KV)	Temperature (°C)	Humidity (%)
0:100	12 w/v	8–9	20–25	40–60
30:70	10 w/v	13–14	20–25	40–60
50:50	10 w/v	14–15	20–25	40–60
70:30	10 w/v	15–16	20–25	40–60

### 2.3 Membrane Characterization

Membrane morphologies were observed by scanning electron microscopy (SEM; JSM-5600LV, JEOL, Japan) at an acceleration voltage of 10 kV. Prior to imaging, the membranes were sputter-coated with Pt for 60 s to increase their conductivity. Fiber diameters and pore sizes were determined from SEM images.

Attenuated total reflection Fourier transform infrared (FT-IR) spectra were recorded on an FT-IR spectrometer (Nicolet-Nexus 670, Thermo Fisher Scientific, United States) using a scan range of 500–4,000 cm^−1^ and a resolution of 2 cm^−1^.

Membrane surface hydrophilicity/hydrophobicity was evaluated by a video contact angle analyzer (Attension Theta, Biolin Scientific AB, Finland). Briefly, deionized water droplets (3 μL) were automatically dispensed onto the membrane surface, and the dynamic changes in their shape were recorded.

Given that the membranes would be applied in a moist environment at 37°C *in vivo*, their post-implantation shrinkage behavior was tested by immersing 1.5 cm × 1.5 cm membrane pieces with attached aluminum foil into physiological saline at 37°C. After 24 h, the membranes were removed and imaged using a high-resolution camera (EOS 6D, Canon, Japan). For post-implantation mechanical property evaluation, the membranes were cut into 5 cm × 1 cm pieces, immersed into physiological saline at 37°C for 24 h as well, loaded on a biomechanical testing machine (Instron-3343, Norwood, United States), and stretched as previously described (Feng et al., 2020).

### 2.4 *In vitro* Evaluation of Membrane Biocompatibility

#### 2.4.1 Isolation and Culturing of Neonatal Rat Ventricular Cardiomyocytes and Cardiac Fibroblasts

Given the membranes were designed for implantation *in vivo* as pericardial substitutes, cardiomyocytes and cardiac fibroblasts were selected as seeding cells to evaluate the biocompatibility of membranes. Cells were isolated from neonatal Sprague Dawley (SD) rats within 3 days after birth using a neonatal rat/mouse cardiomyocyte isolation kit. Briefly, neonatal SD rats were sterilized with 75 vol% aqueous ethanol, the chests were opened, hearts were removed, and ventricular tissues were cut and digested in a specific enzyme buffer for 12 min at 37°C. Then, the supernatant was transferred to a new tube, and new enzyme buffer was added to digest the remaining tissues. This step was repeated four to six times until all the tissues have been digested. Finally, the supernatant was collected and centrifuged at 1,200 rpm for 1 min to afford the desired cells as a pellet. After 2-h culturing, most cardiac fibroblasts were attached to the bottom of the plates, and the unattached cells were transferred to new plate and cultured for another 4 h to remove residual cardiac fibroblasts and afford cardiomyocytes suspended in the culture medium. The two types of cells were cultured in DMEM/F-12 medium with 10% FBS and 1% penicillin-streptomycin and ready for further experiments after 48 h.

#### 2.4.2 Evaluation of Membrane Cytotoxicity

Nanofibrous membranes were collected on circular cover slices (diameter = 15 mm), exposed to ultraviolet radiation for 30 min in a biological safety cabin, placed at the bottom of a 24-well plate with steel rings, and pre-cultured in DMEM/F-12 medium with 10% FBS and 1% penicillin-streptomycin overnight. In view of the rapid proliferation capacity of cardiac fibroblasts, cardiomyocytes and cardiac fibroblasts were seeded onto the membranes at densities of 5 × 10^4^ and 2.5 × 10^4^ cells per well, respectively. Cell viability was determined using the live and dead cell viability/cytotoxicity assay kit after one and 7 days of culturing. Briefly, the cells were incubated in with 2 μM calcein AM and 4 μM ethidium homodimer-1 for 1 h in the dark, washed in Dulbecco’s phosphate buffered saline three times, and directly imaged using a laser confocal microscope system (TSC SP8, Leica, Germany).

#### 2.4.3 Efficiency of Cell Seeding on Membranes

Cardiomyocytes and cardiac fibroblasts were counted using an automated cell counter (iM1200, Countstar, China) to determine the total cell number and total live cell number prior to seeding. Given their absolutely different adhesion speeds, cardiac fibroblasts were incubated for 6 h, while cardiomyocytes overnight. At predetermined times, the culture medium was collected to determine the total unattached cell number suspended in medium by the automated cell counter again. Hence, the total attached cell number = total cell number − total unattached cell number. The seeding efficiency (%) was calculated as 100% × total attached cell number/total live cell number.

#### 2.4.4 Morphology of Cells on Membranes

The morphology of membrane-attached cells was observed using immunostaining. After one- and 5-day incubation on membranes, cells were fixed with 4% paraformaldehyde for 30 min, permeabilized with 0.5% Triton X-100 for 10 min and blocked with 10% goat serum in phosphate-buffered saline (PBS) for 1 h at room temperature. Two primary antibodies (anti-cardiac troponin T and anti-vimentin antibodies for cardiomyocytes and cardiac fibroblasts, respectively) were used according to manufacturer’s instructions. Additionally, the antibodies were labeled with fluorescent conjugated secondary antibodies for another 2 h, and the cell nuclei were stained with DAPI for 30 s in the dark. The samples were washed three times with PBS on a shaker and visualized using a confocal microscope.

#### 2.4.5 Cell Proliferation on Membranes

Cell proliferation on membranes was evaluated using CCK-8 on days one, three, five, and seven. Prior to measurements, the cells were cultured in another medium containing 10% CCK-8 for 2.5 h. Absorbance was measured at 450 nm using a microplate reader (Multiskan MK3, Thermo Electron Corporation, United States).

### 2.5 *In vivo* Evaluation of Membrane Anti-Adhesion Efficacy

#### 2.5.1 Animals

Three-month-old healthy male New Zealand white rabbits weighing 2–2.5 kg were obtained from Shanghai Jiaotong University Agricultural Experimental Practice Field (Shanghai, China), housed in a temperature-controlled room (22°C) and fed a standard laboratory diet and water. All animal experiments were approved by the Animal Care and Experiment Committee of Shanghai Jiaotong University Agricultural Experimental Practice Field. The experiment was set up consisting five groups (positive control, 0:100, 30:70, 50:50, and 70:30) at 1 month after surgery, each group was analyzed for 5 animals. And at another time point of three months, positive control (6 animals), 0:100 (8 animals which considered its strong individual differences one month after surgery), 30:70 (6 animals), and 50:50 (6 animals) were analyzed, while two animals were died after surgery (one positive control group and one 0:100 group). In total, 51 animals were used in this study.

#### 2.5.2 Surgical Procedures

The animals were divided into one positive control group and four experimental groups (GT:PCL = 0:100, 30:70, 50:50, and 70:30). In this study, we optimized our previous experimental procedure (e.g., cut the membranes into 1.5 cm × 1.5 cm pieces) and paid more attention to postoperative treatment and ethical care. Cefuroxime (30 mg/kg) was intramuscularly administered for anti-infection for three consecutive days, while tramadol (50 mg) was intramuscularly administered for analgesia for two consecutive days. Other specific surgical protocols were almost consistent with those used previously (Feng et al., 2019).

#### 2.5.3 Heart Function Evaluation

Echocardiography was used one and 3 months after surgery to determine whether the nanofibrous membranes influenced heart function or induced ventricular remodeling. Briefly, the rabbits were anesthetized by intravenous injection of sodium pentobarbital (30 mg/kg), and transthoracic echocardiography was performed from the right sternum using a 12S probe (Vivid E95, GE, United States) to detect the long axial section of the left ventricle. The left ventricular ejection fraction (LVEF) and fractional shortening (LVFS) required indirect calculation, while the left ventricular posterior wall thickness (LVPWd) and interventricular septal thickness (IVSd) at end-diastole were directly determined using M-mode echocardiography. Six healthy rabbits were randomly selected for detection before surgery as a normal control.

#### 2.5.4 Liver/Kidney Function and CRP/Immunoglobulin Level Assessments

The liver/kidney function and CRP/immunoglobulin levels were evaluated one and 3 months after surgery to assess the biosafety of membranes after implantation. Seven (aspartate transaminase (AST), alanine transaminase (ALT), total bilirubin (TBIL), total protein (TP), albumin (ALB), globulin, albumin/globulin (A/G)) and three (blood urea nitrogen (BUN), creatinine (CREA), and cystatin C (Cys C)) commonly used indices were employed to assess liver and kidney functions (Chen et al., 2015a), respectively. Four immunoglobulins (IgG, IgM, IgA, and total IgE) were selected to evaluate the immune rejection level (Jin et al., 2019). Specifically, ∼3 ml of fasting blood was collected from the internal jugular vein after completing the echocardiography. 2-ml blood was placed into a non-coagulated sterile tube and centrifuged at 4,000 rpm for 10 min. The upper serum was analyzed to determine the liver/kidney function and immunoglobulin levels using an automatic biochemical analyzer (C16000, Abbott Architect, United States) and a specific protein analyzer (BN II, Siemens, Germany), respectively. The remaining 1 ml was promptly transferred to an ethylene diamine tetraacetic acid (EDTA) blood collection tube, and the CRP level was determined by an automated specific protein iPOCT workstation (Ottoman-1000, Ottoman, China). All procedures were performed in strict accordance with manufacturer’s instructions. Given the short half-life of immunoglobulins (maximal at ∼28 days for IgG), their levels were examined only for a period of 1 month after surgery. The basic normal levels of liver/kidney function, CRP and immunoglobulins were determined by six healthy rabbits before surgery as well.

#### 2.5.5 Overall Observation

Following echocardiography and blood collection, each rabbit was euthanized with sufficient sodium pentobarbital and underwent repeated sternotomy. The macroscopic adhesions were scored by an experienced surgeon, who was blinded for the purpose of this study, and the adhesion was scored using a previously reported standardized scale (Malm et al., 1992): 0 = no adhesion between the heart and sternum/material; 1 = mild adhesions, easy to separate by blunt dissection; 2 = moderate adhesions, partially requiring sharp dissection; 3 = severe adhesions, mainly requiring sharp dissection and easy bleeding. Another blinded observer assisted in image acquisition at the same time.

#### 2.5.6 Histological Staining

After overall observation, the membranes were carefully removed from surrounding tissues, fixed in 10% neutral buffered formalin for at least 24 h, dehydrated using alcohol gradients, embedded in paraffin, and sectioned into 5-μm-thick slices for H-E staining to assess the inflammatory reaction and physical barrier function.

### 2.6 Statistical Analysis

All quantitative data were presented as means ± standard deviations. One-way analysis of variance followed by Tukey’s post hoc test was used to determine statistically significant differences between groups. Significant difference was considered at *p < 0.05; **p < 0.01; ***p < 0.001.

## 3 Results

### 3.1 Preparation and Characterization of GT/PCL Membranes

**FIGURE 1:**
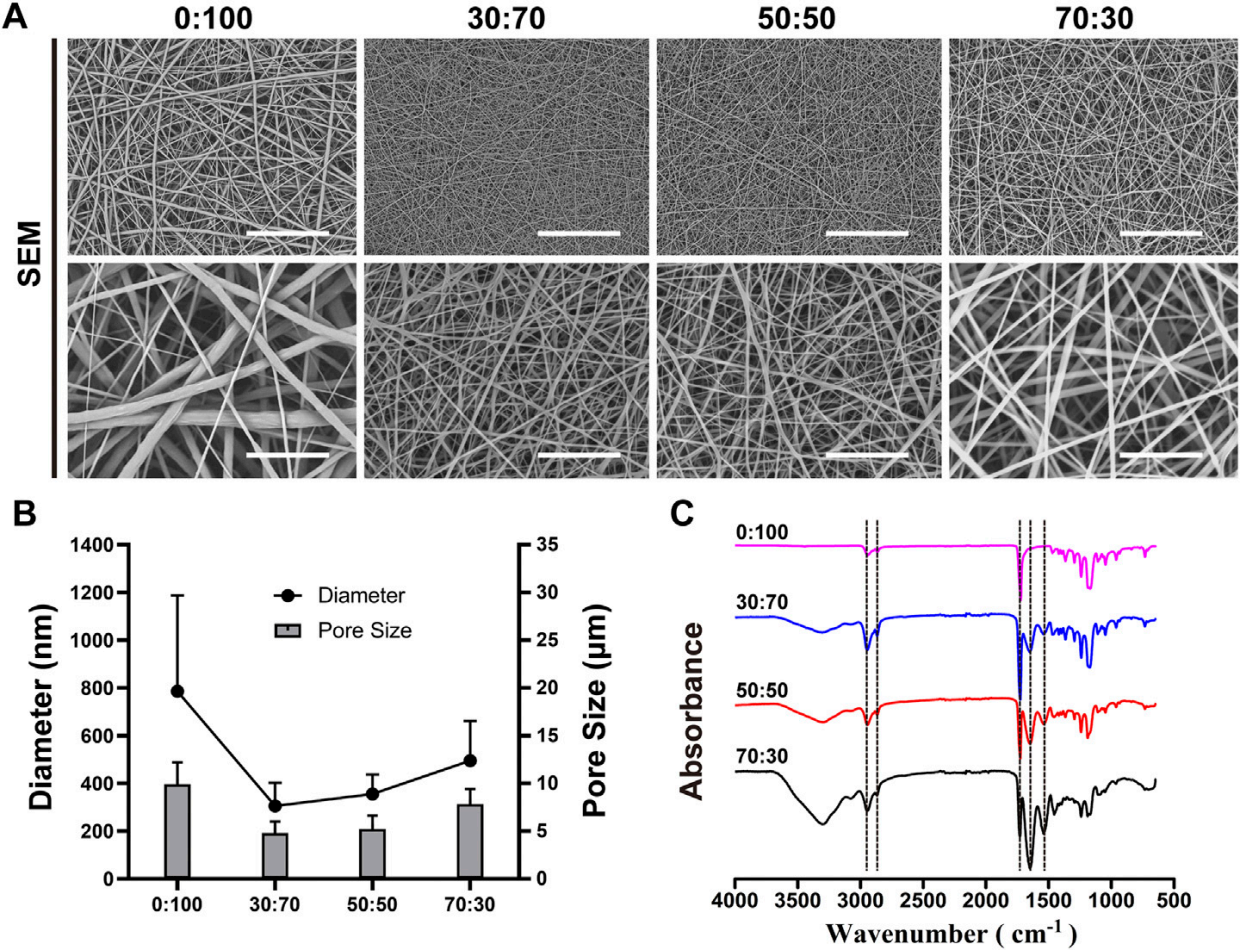
Representative **(A)** SEM micrographs [scale bars equal 50 μm (low magnification) and 10 μm (high magnification)], **(B)** fiber diameters and pore sizes, and **(C)** FT-IR spectra of nanofibrous membranes.

As shown in Figure 1A, all fibers exhibited a smooth surface without beads or bonding. The pure-PCL (0:100) membrane fibers were relatively thick (average diameter = 785.5 ± 401.5 nm), while thinner fibers were present in GT-containing membranes (average diameters = 304.7 ± 98.1 and 355.7 ± 81.0 nm for GT:PCL = 30:70 and 50:50, respectively). However, at a ratio of 70:30, the fiber diameter increased to 494.7 ± 166.6 nm. As for the pore size, which is an important parameter for selection of membranes as barriers to prevent cell penetration, increased with the increasing fiber diameter (Figure 1B), equaling 9.9 ± 2.3, 4.8 ± 1.2, 5.2 ± 1.4, and 7.8 ± 1.6 µm for different ratios of 0:100, 30:70, 50:50, and 70:30, respectively. Membrane composition was probed by FT-IR (Figure 1C). The characteristic peaks of PCL at 2,943 cm^−1^ (asymmetric CH_2_ stretch), 2,865 cm^−1^ (symmetric CH_2_ stretch), and 1,724 cm^−1^ (C=O stretch) were observed for all membranes, while the characteristic peaks of GT at 1,652 cm^−1^ (amide I) and 1,540 cm^−1^ (amide II) were only observed for GT/PCL membranes. The amide I band was attributed to the random coil and α-helix conformations of GT. As expected, the areal fractions of PCL- and GT-specific peaks decreased and increased with increasing GT content, respectively.

**FIGURE 2:**
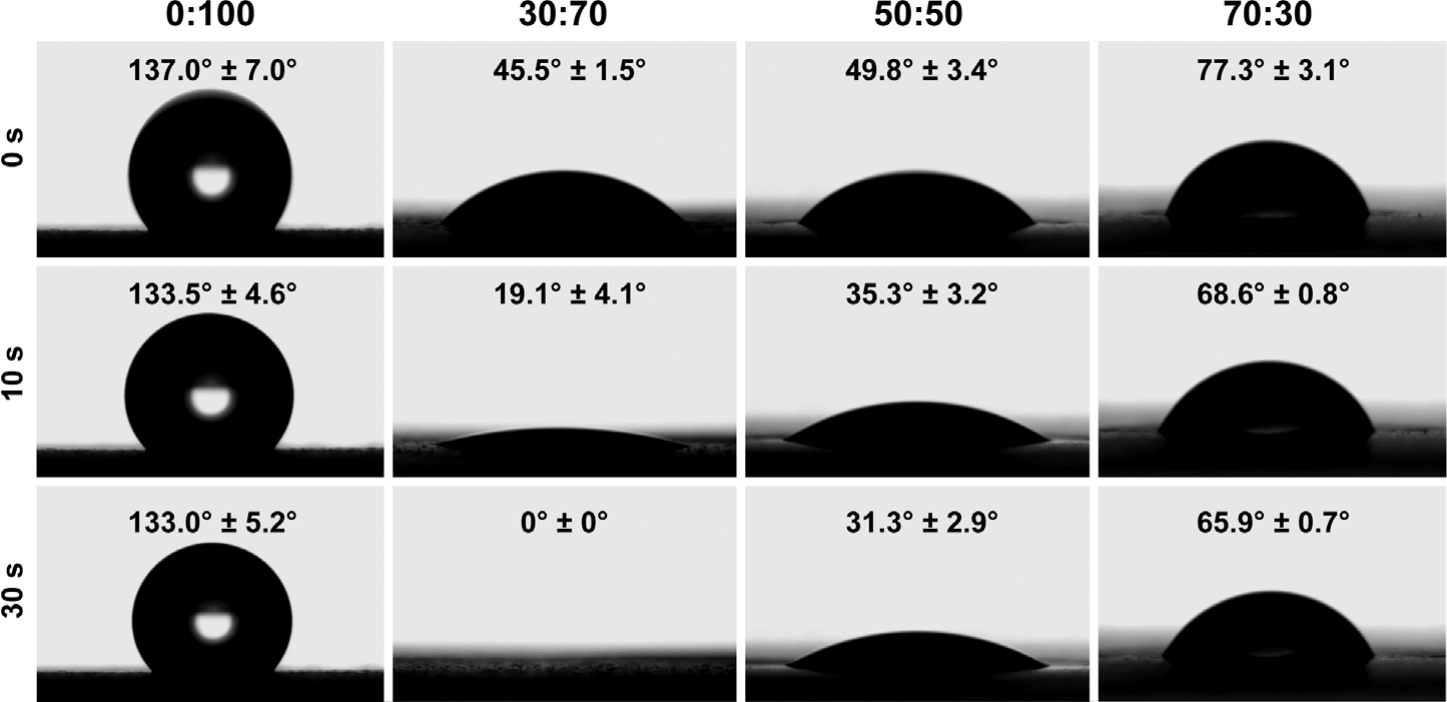
Water contact angles of different nanofibrous membranes.

The 0:100 membrane was highly hydrophobic, and the water droplets deposited thereon formed a large obtuse contact angle (137.0 ± 7.0°) and remained stable. The incorporation of GT greatly increased hydrophilicity, and all GT/PCL membranes presented hydrophilic surfaces with acute contact angles (Figure 2). In the case of the 30:70 membrane, water was rapidly absorbed to afford a contact angle of zero within 30 s. However, the rate of water absorption decreased at higher GT contents, in line with the fact that water absorption by pure-GT membranes is also insufficient (Feng et al., 2012).

**FIGURE 3:**
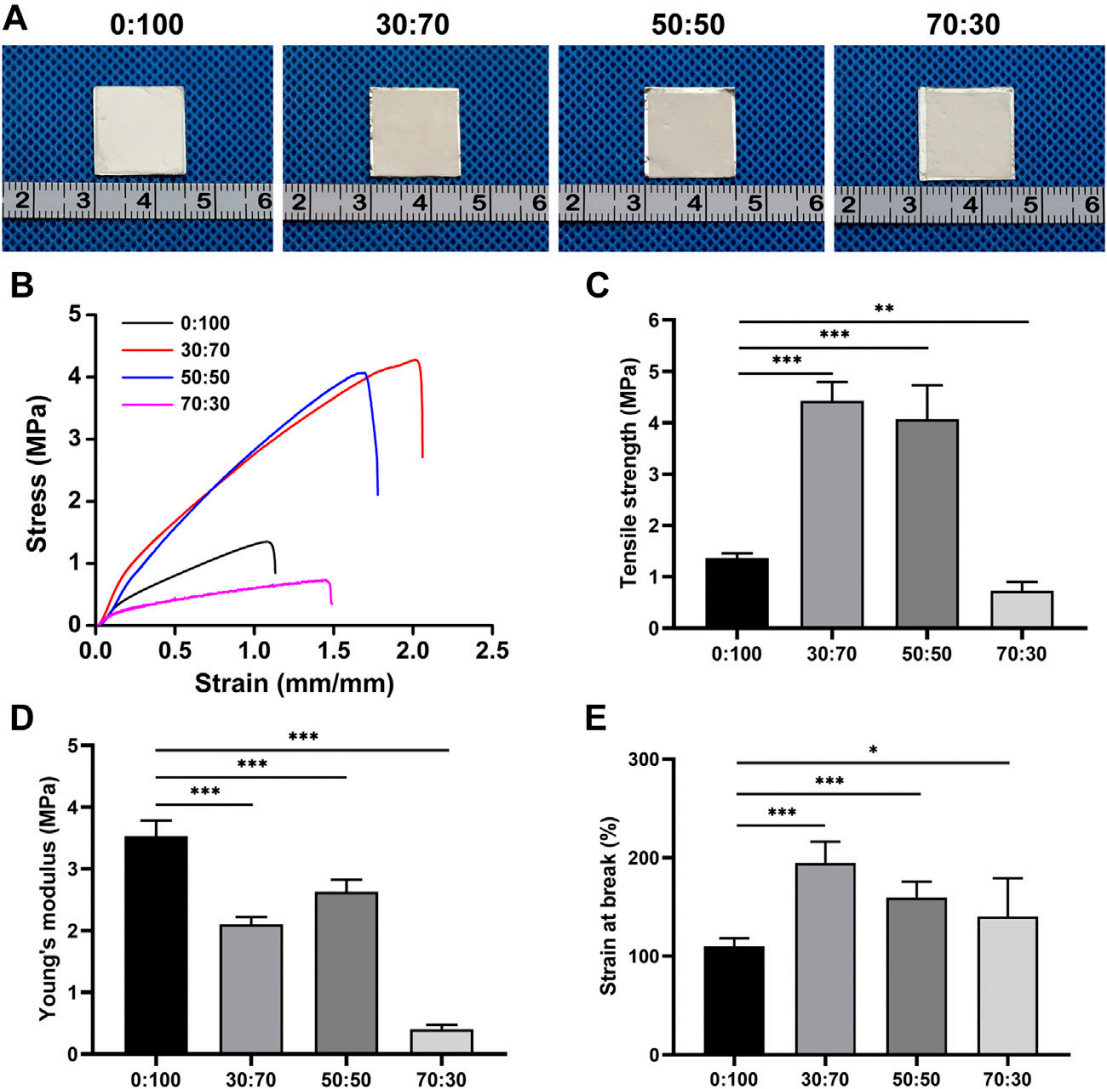
**(A)** Gross views, **(B)** stress-strain curves, **(C)** tensile strengths, **(D)** Young’s modulus, and **(E)** strains at break of membranes after 24-h immersion into physiological saline at 37°C (*p < 0.05; **p < 0.01; ***p < 0.001).

As the membranes were implanted *in vivo*, they were immersed into saline held at 37°C to characterize shrinkage under simulated physiological conditions. Compared with their original size (i.e., the size of the underlying aluminum foil), the 0:100 membrane did not shrink, while 30:70, 50:50, and 70:30 membranes slightly contracted, which indicated that all membranes had good thermal stability (Figure 3A). However, these membranes showed different mechanical properties (Figure 3B; Table 2). The introduction of GT greatly improved the mechanical properties of membranes with the increased maximum tensile strength (Figure 3C) and strain at break (Figure 3E), while significant mechanical property deterioration was observed at a GT content of 70%, as exemplified by the decreased maximum tensile strength (Figure 3C) and Young’s modulus (Figure 3D). In particular, the maximum tensile strength decreased in the order of 4.4 ± 0.4 MPa (30:70) > 4.1 ± 0.6 MPa (50:50) > 1.4 ± 0.1 MPa (0:100) > 0.7 ± 0.2 MPa (70:30).

**TABLE 2:** Mechanical parameters calculated from the strain-stress curves of wet membranes.

Membrane	Young’s modulus (MPa)	Tensile strength (MPa)	Strain at break (%)
0:100	3.5 ± 0.2	1.4 ± 0.1	110.0 ± 8.1
30:70	2.1 ± 0.1	4.4 ± 0.3	194.6 ± 21.4
50:50	2.6 ± 0.2	4.1 ± 0.7	159.4 ± 16.2
70:30	0.4 ± 0.1	0.7 ± 0.2	140.3 ± 39.0

### 3.2 Biocompatibility of GT/PCL Membranes *in vitro*

**FIGURE 4:**
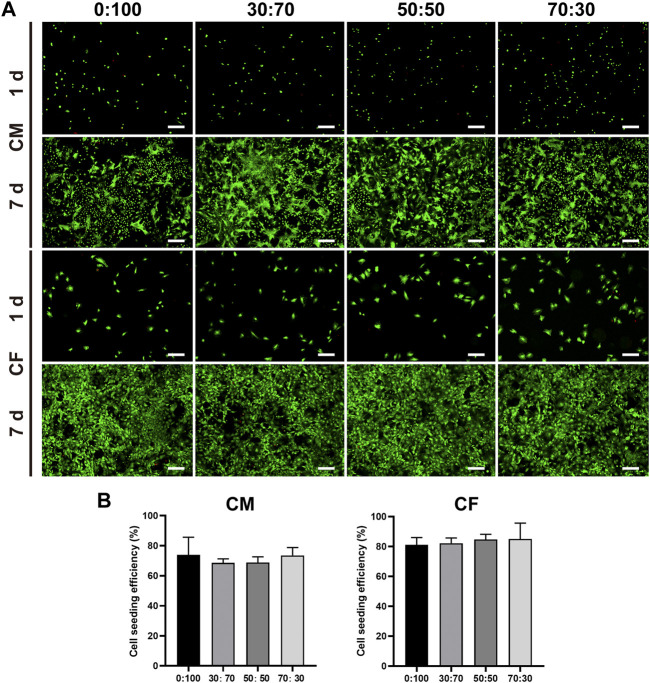
**(A)** Representative live and dead staining images of cardiomyocytes and cardiac fibroblasts cultured on different membranes for one and 7 days. Scale bars = 200 μm. **(B)** Seeding efficiencies of cardiomyocytes and cardiac fibroblasts on different membranes.

The viability of cardiomyocytes and cardiac fibroblasts on membranes was assessed using the live/dead cell assay. In the obtained images, green fluorescence represents live cells, while red fluorescence represents the dead. After 1 day of culturing, both cardiomyocytes and cardiac fibroblasts adhered well to all membranes, as exemplified by the numerous green dots (live cells) and few red dots (dead cells) (Figure 4A). The cells continued to grow with time, reaching ∼90% (cardiomyocytes) and 100% (fibroblasts) confluency on all membranes with bright green fluorescence on day seven. In addition, cell seeding efficiencies reached ∼70% (cardiomyocytes) and 80% (fibroblasts) on all membranes without significant differences (Figure 4B).

**FIGURE 5:**
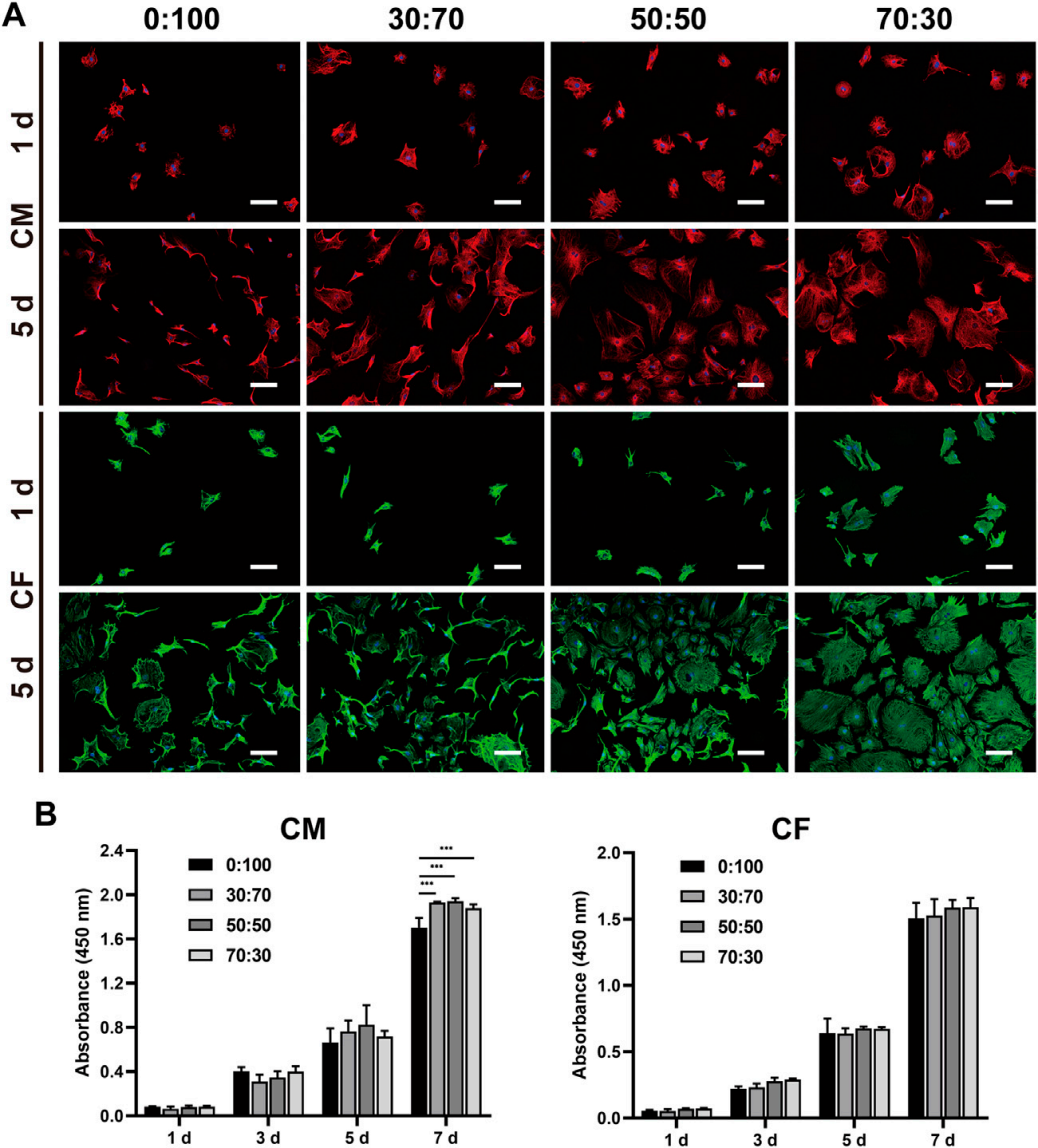
**(A)** Representative immunofluorescence staining images of cardiomyocytes and cardiac fibroblasts cultured on different membranes for one and 5 days. Blue: nucleus. Red: cTNT. Green: vimentin. Scale bars = 100 μm. **(B)** Proliferation of cardiomyocytes and cardiac fibroblasts cultured on different nanofibrous membranes for one, three, five, and 7 days. (***p < 0.001).

The morphology and proliferation of cells cultured on membranes were evaluated using immunofluorescence staining (Figure 5A). Troponin T (cardiomyocytes) and vimentin (fibroblasts) staining showed that both cells well adhered and spread on all membranes on day one, although a larger fibroblast spreading area was observed for the 70:30 group. With increasing cultivation time, cardiomyocytes and fibroblasts rapidly proliferated on all membranes at day five. In line with the results of immunofluorescence staining, those of the CCK-8 assay (Figure 5B) revealed that cardiomyocytes and fibroblasts exhibited steady and continuous proliferation without significant difference between groups, except for the downward trend in the 0:100 group at day seven for cardiomyocytes.

Collectively, these results indicated that all GT/PCL membranes had good biocompatibility with cardiomyocytes and cardiac fibroblasts and were suitable for applications in cardiac surgery.

### 3.3 Anti-adhesion Efficacies of GT/PCL Membranes One Month After Surgery

**FIGURE 6:**
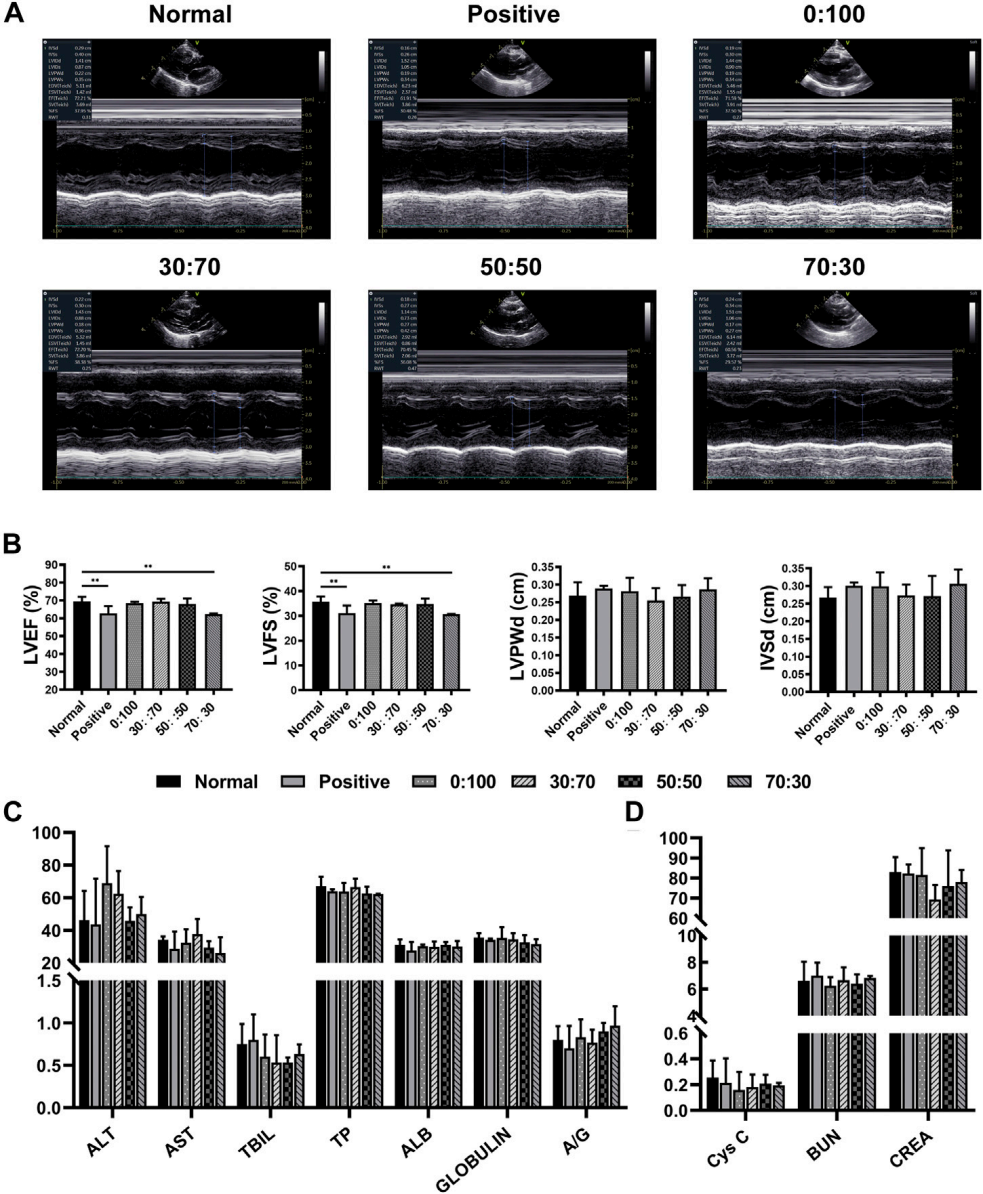
One month after surgery **(A)** representative echocardiography images, **(B)** heart function and **(C,D)** liver/kidney function for normal and experimental groups. (**p < 0.01).

One month after surgery, the heart/liver/kidney functions and CRP/immunoglobulin lever assessments were used to detect whether the membranes would cause adverse reactions *in vivo* implantation. Echocardiography (Figures 6A,B) showed that compared with the normal group (healthy rabbits that did not undergo surgery), the heart function indicators LVEF and LVFS in the positive control and 70:30 groups were significantly reduced, whereas no significant differences were observed among the 0:100, 30:70, and 50:50 groups. Conversely, there were no significant differences in liver (Figure 6C) or kidney (Figure 6D) function indicators among all groups, which suggested that the membranes were not toxic to the liver and kidneys. Additionally, the results of CRP and immunoglobulin level evaluation (Table 3) were not significantly different among all groups either, indicating that the membranes did not induce strong inflammation reaction and immune rejection response after implantation *in vivo*. Collectively, these results demonstrated that all the membranes exhibited sufficient biosafety for implantation *in vivo*, except for the 70:30 group, in which case a reduction in heart function (i.e., in LVEF and LVFS) was observed.

**TABLE 3:** CRP and immunoglobulin levels in different groups 1 month after surgery.

Indicator	Normal	Positive	0:100	30:70	50:50	70:30
CRP (mg/L)	<1	<1	<1	<1	<1	<1
IgG (g/L)	<0.07	<0.07	<0.07	<0.07	<0.07	<0.07
IgA (g/L)	<0.07	<0.07	<0.07	<0.07	<0.07	<0.07
IgM (g/L)	<0.18	<0.18	<0.18	<0.18	<0.18	<0.18
Total IgE (IU/ml)	<4.23	<4.23	<4.23	<4.23	<4.23	<4.23

**FIGURE 7:**
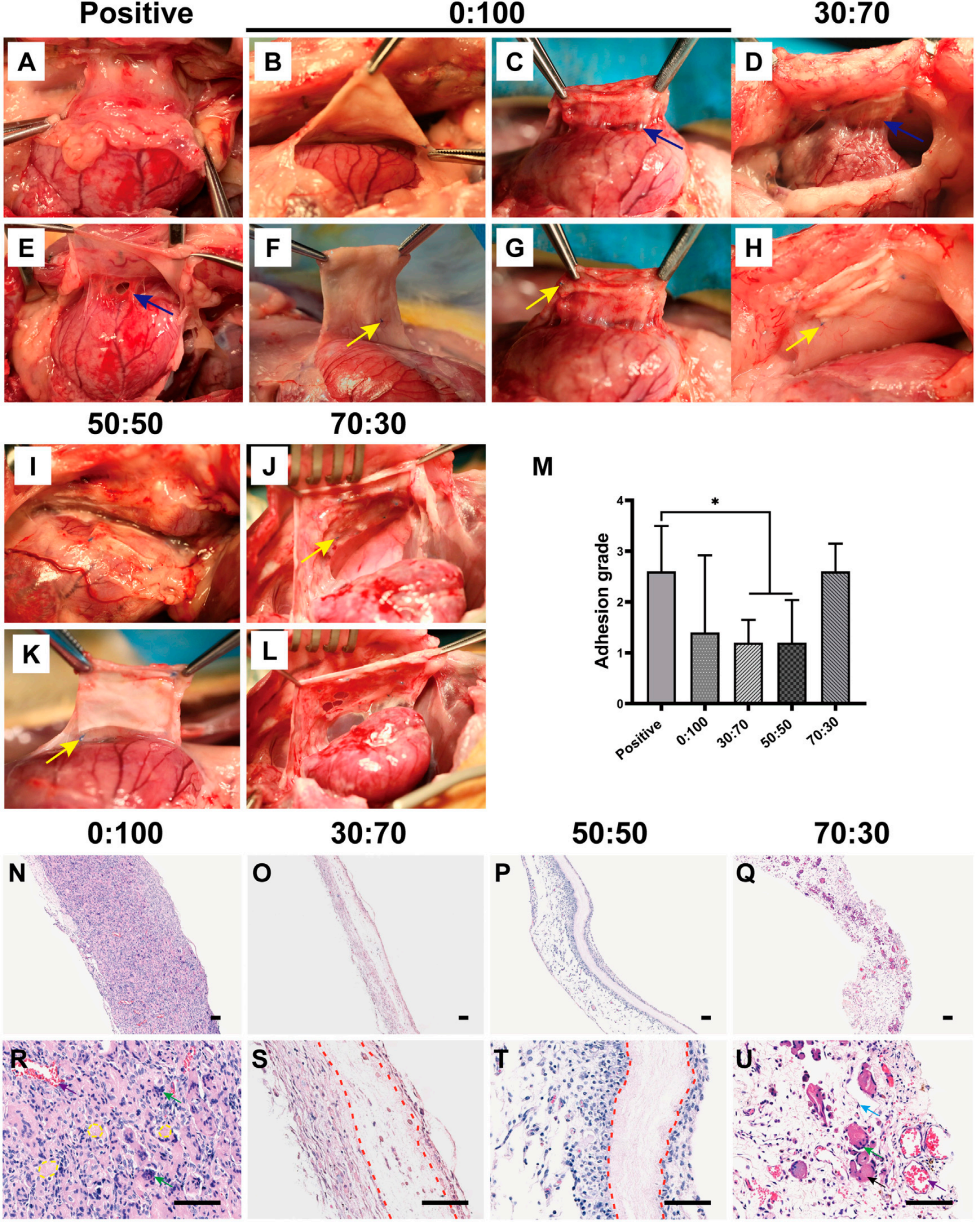
One month after surgery representative overall images for **(A,E)** positive control, **(B,C,F,G)** 0:100, **(D,H)** 30:70, **(I,K)** 50:50, and **(J,L)** 70:30 groups, blue arrows indicate adhesion sites, and yellow arrows show sutures positions. **(M)** pericardial adhesion grades, *p < 0.05, and representative H-E staining images for **(N,R)** 0:100, **(O,S)** 30:70, **(P,T)** 50:50, and **(Q,U)** 70:30 groups, yellow circles indicate “islands,” red dot lines indicate “barriers.” Green, blue, black, and purple arrows show multinucleated giant cells, fibroblasts, muscular tissues, and blood vessels, respectively. Scale bars = 80 μm.

After echocardiography and blood examination, animals were anesthetized and sacrificed. The thoracic cavity was reopened, and adhesions between the heart and sternum were separated by dissection. In the positive control group, severe adhesions were formed at the injury site of the previous operation (Figures 7A,E), and more attention had to be paid during dissection to keep the heart intact. Strong individual differences were observed in the 0:100 group, i.e., some rabbits had no or slight adhesions that could be easily separated by blunt dissection (Figures 7B,F); whereas others had thick and heavy adhesions requiring continuous sharp dissection (Figures 7C,G). However, mild filamentous adhesions were observed in 30:70 (Figures 7D,H) and 50:50 (Figures 7I,K) groups, the membranes could be easily separated from the heart and maintained their original shape with a white appearance. In the 70:30 group, intense and solid adhesions were observed, and the original membrane shape was hardly recognizable due to the rapid degradation of GT (Figure 7J). Broken holes and bleeding were easily induced upon membrane detachment from the sternum due to the poor mechanical properties of this membrane (Figure 7L). Furthermore, according to the results of adhesion grade scoring (Figure 7M), the adhesions were severe in the positive control (score = 2.6 ± 0.9) and 70:30 (2.6 ± 0.5) groups. Much lower scores were observed for 30:70 (1.2 ± 0.4) and 50:50 (1.2 ± 0.8) groups, while a very large score fluctuation was observed for the 0:100 group (1.4 ± 1.5).

After overall observation, the membranes were carefully removed from surrounding tissues. Histological analysis demonstrated that though all the membranes had the same thickness before surgery, the thickness of 0:100 membrane significantly exceeded than others after implantation. Numerous inflammatory cells have entirely infiltrated into the 0:100 membrane regardless of whether adhesions were slight or strong (Figure 7N). High-magnification imaging showed that the membrane was divided into masses of small islands, many macrophages gathered and ranged along islands to form multinucleated giant cells thereby inducing membrane disintegration and phagocytosis (Figure 7R). In the 30:70 and 50:50 groups, obvious pink barriers were observed with several (30:70, Figures 7O,S) or no (50:50, Figures 7P,T) infiltrating cells and a small number of inflammatory cells and sporadic blood capillaries distributed on both sides of barriers. While in the 70:30 group, many monocytes, multinucleated giant cells, fibroblasts, blood vessels and muscular tissues have invaded into the membranes, and no barriers were formed (Figures 7Q,U).

Considering the 70:30 membrane was not only difficult to suture during surgery but also could not maintain its structural integrity to prevent cardiac postoperative adhesion after being implanted *in vivo*, and therefore, this membrane was not investigated further.

### 3.4 Anti-adhesion Efficacies of GT/PCL Membranes Three Months After Surgery

**FIGURE 8:**
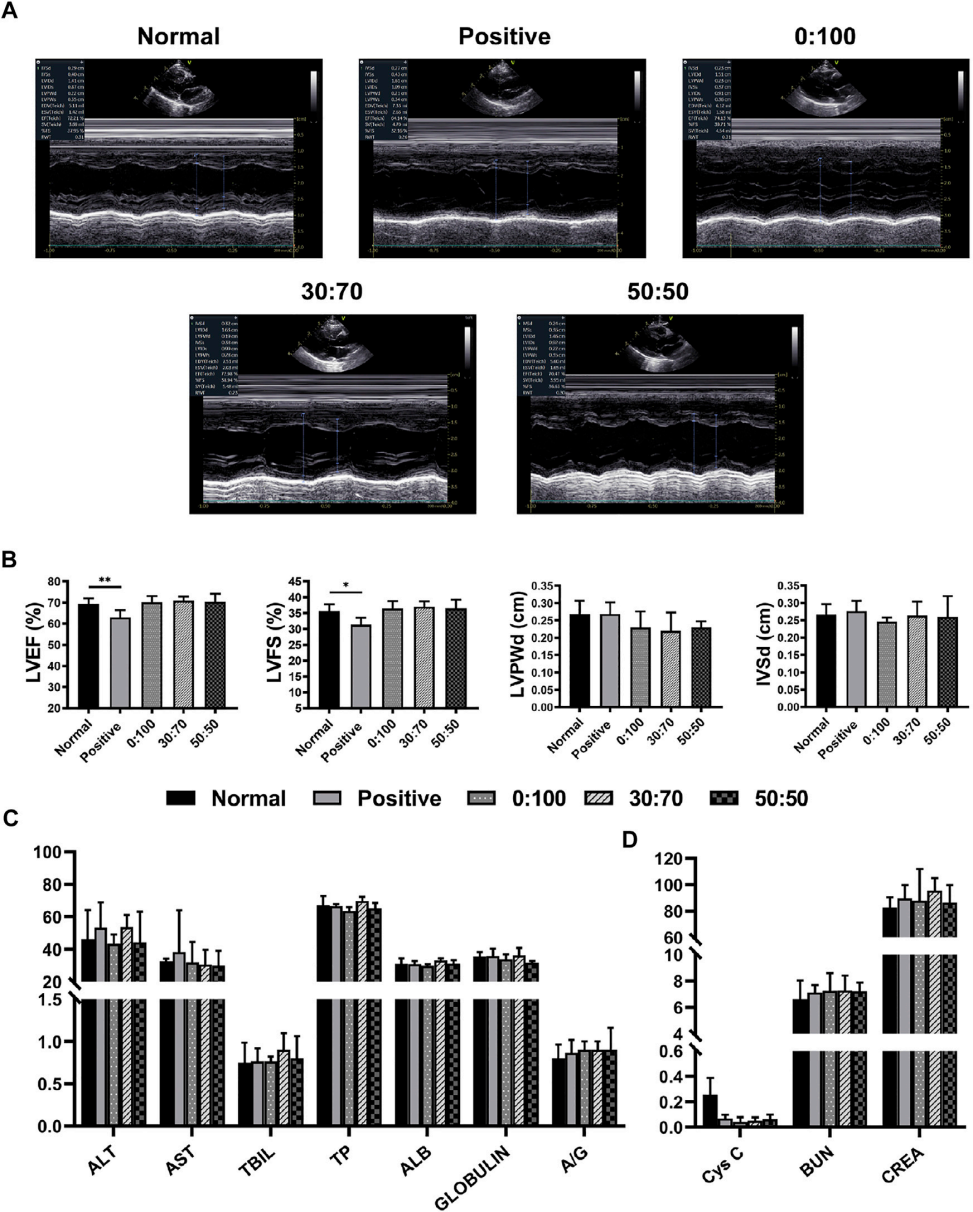
Three months after surgery **(A)** representative echocardiography images, **(B)** heart function and **(C,D)** liver/kidney function for normal and experimental groups. (*p < 0.05; **p < 0.01).

Echocardiography (Figures 8A,B) showed that compared with the normal group, the heart function (i.e., LVEF and LVFS values) was significantly reduced in the positive control group, while no significant differences were observed for other three groups (0:100, 30:70, and 50:50). Further blood examination showed that the liver/kidney function indicators (Figures 8C,D) of normal group were not significantly different from those of other groups, and CRP levels were still less than 1 mg/L for all groups. These results were consistent with those obtained 1-month after surgery, which illustrated the high biosafety of PCL and GT/PCL (30:70 and 50:50) membranes for long-term *in vivo* implantation.

**FIGURE 9:**
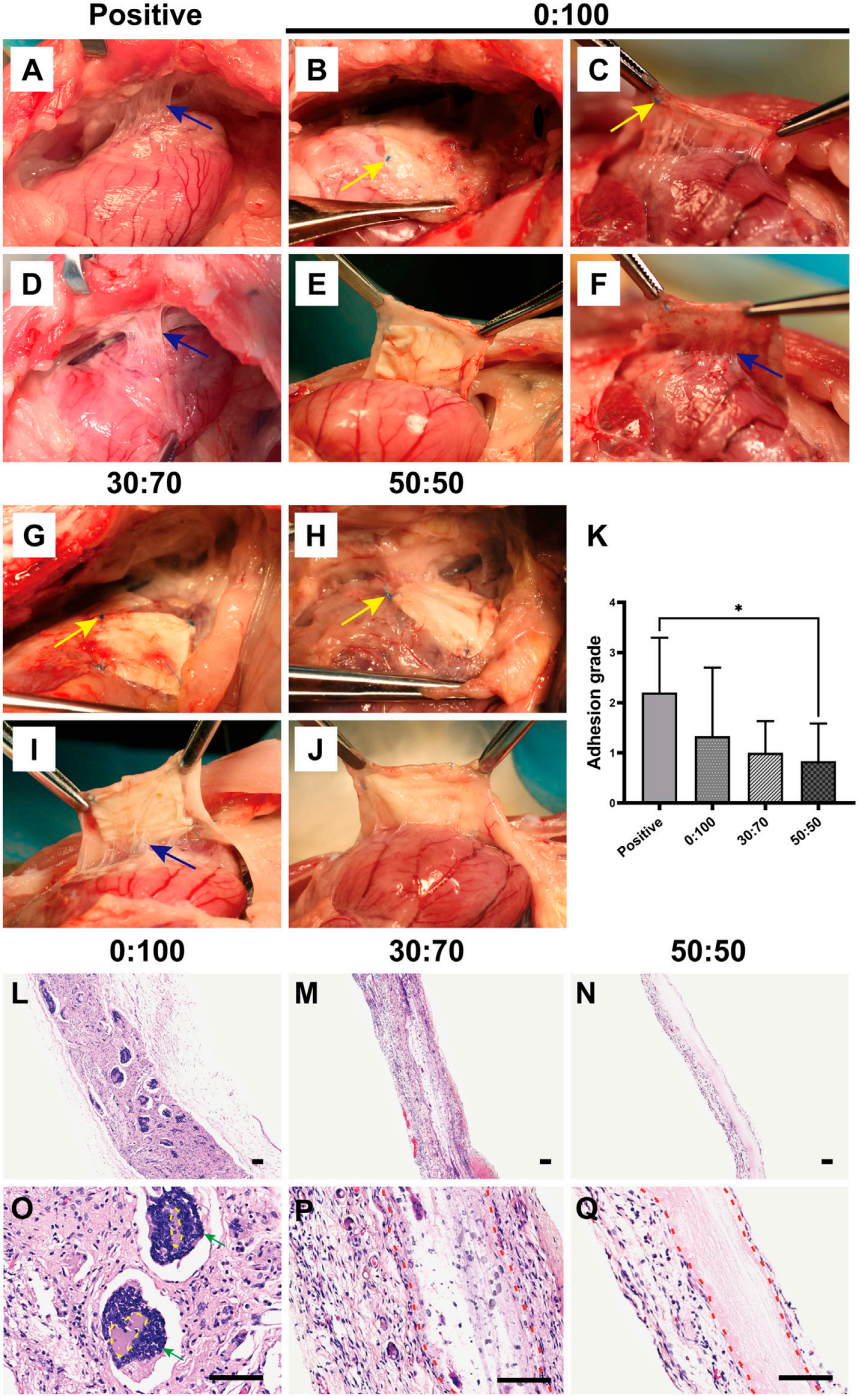
Three months after surgery representative overall images for **(A,D)** positive control, **(B,C,E,F)** 0:100, **(G,I)** 30:70, and **(H,J)** 50:50 groups, blue arrows indicate adhesion sites, and yellow arrows show sutures positions. **(K)** Pericardial adhesion grades, *p < 0.05, and representative H-E staining images for **(L,O)** 0:100, **(M,P)** 30:70, and **(N,Q)** 50:50 groups, yellow circles indicate “islands,” red dot lines indicate “barriers,” green arrows indicate multinucleated giant cells. Scale bars = 80 μm.

Overall, dense and solid adhesions were observed between the pericardial defect area and sternum in the positive control group (Figures 9A,D). These adhesions were more serious than those formed 1-month after surgery and could only be separated by sharp dissection. And the adhesion conditions still showed large variations between individual animals in the 0:100 group, i.e., some rabbits had no or slight adhesions (Figures 9B,E), while the others had severe adhesions (Figures 9C,F). However, filamentous and sparse adhesions were observed between the membranes and epicardium in 30:70 (Figures 9G,I) and 50:50 (Figures 9H,J) groups, which could be easily separated by digital dissection. Specifically, the membrane became blurred and developed a yellow-like appearance similar to that of the native pericardium tissues. The heart was smooth and moist, and the coronary arteries were clearly visible. The related adhesion grade scores were shown in Figure 9K. The adhesion was strongest in the positive control group (score = 2.2 ± 1.1), with lower scores observed for 30:70 (1.0 ± 0.6) and 50:50 (0.8 ± 0.8) groups. As in the case of 1 month after surgery, a large standard deviation was observed for the 0:100 group (1.3 ± 1.4).

Histological staining showed that the 0:100 membrane gradually degraded, and most islands have disappeared (Figure 9L). Multinucleated giant cells were much bigger and more visible than 1 month after surgery, forming many dark purple (nucleus) bumps (Figure 9O). In the 30:70 group, some inflammatory cells together with small arteries and blood capillaries infiltrated the membrane, and the barrier became blurred (Figures 9M,P). However, an obvious pink barrier with no cell infiltration was observed in the 50:50 group (Figures 9N,Q), indicating that this membrane could act as a perfect barrier to prevent cell infiltration, which was in line with the results obtained 1 month after surgery.

## 4 Discussion

Adhesion is one of the most frequent postoperative complications with many serious consequences. For example, peritoneal adhesions are commonly observed after abdominal and pelvic surgery, leading to chronic abdominal pain, intestinal obstruction, and female infertility (Hol et al., 2019; Tang et al., 2020). And pelvic adhesions resulting from gynecologic surgery may also cause chronic pelvic pain, small bowel obstruction, and female infertility (Al-Jabri and Tulandi, 2011; Dawood and Elgergawy, 2018). Besides, tendon adhesions not only cause dysfunction and pain, but also usually require surgical intervention to loosen (Liu et al., 2013; Cai et al., 2021). In addition, many patients undergoing cardiac surgery require reoperation even multiple operations. Owing to the adhesions generated by the first operation, original anatomical layers and gaps have been disappeared, leaving the reoperation a daunting task. In our pilot study, electrospun membranes with a GT:PCL mass ratio of 50:50 have showed high potential in reducing sternal and epicardial adhesions after cardiac surgery (Feng et al., 2019). However, the GT:PCL ratio can affect the physical structure, mechanical properties, degradation rate, and biocompatibility of membranes, which may eventually lead to changes in anti-adhesion efficacy. Thus, GT/PCL membranes with various compositions were investigated herein.

Good mechanical properties are a primary prerequisite for given materials used as anti-adhesion barriers after cardiac surgery, as the membranes must withstand the stretching during surgical suturing and the forces generated during movements *in vivo*. PCL is a synthetic bioresorbable polymer with excellent mechanical properties, while GT is a natural biodegradable polymer in poor mechanical properties. Surprisingly, in line with our previous findings (Feng et al., 2012), the hybridization of PCL with GT (GT:PCL = 30:70 and 50:50, w/w) did not deteriorate but rather enhance the mechanical properties than pure PCL membranes (Figures 3C,E). This behavior was ascribed to the good miscibility of GT and PCL, their good entanglement made the molecular chains difficult to slide under loading. However, at an overly high GT content of 70 wt%, the mechanical properties worsened (Figures 3C,D), which complicated the suture during surgery and then resulted in increasing operation time and difficulties. Further animal dissection demonstrated that this membrane was easily broken, hardly maintained its structural integrity (Figures 7J,L), and could not act as an effective physical barrier to prevent postoperative cardiac adhesion (Figures 7Q,U). It is known that the structural integrity is a critical consideration as a physical barrier. Therefore, the 70:30 membrane was concluded to be unsuitable for preventing postoperative cardiac adhesion because of its poor mechanical properties.

Biocompatibility is another important factor for assessing the suitability of membranes to be implanted *in vivo*. *L*ive/dead staining showed that all the tested membranes had no cytotoxicity to cardiomyocytes and cardiac fibroblasts *in vitro*, and there were no obvious differences on cell seeding efficiencies as well (Figure 4). Furthermore, the cells adhered well and experienced rapid proliferation on all membranes, as revealed by immunofluorescence staining and the CCK-8 assay (Figure 5). *In vivo* studies have shown that heart/liver/kidney functions (Figures 6, 8) and CRP/immunoglobulin levels (Table 3) in 0:100, 30:70, and 50:50 groups were not different from those of normal healthy rabbits. These results indicated that PCL and GT/PCL (30:70 and 50:50) membranes are biosafety enough and did not affect the heart/liver/kidney functions or induce intense inflammation and immune rejection responses after implantation *in vivo*. Given their good mechanical properties and excellent biocompatibility, the 0:100, 30:70, and 50:50 membranes showed promising potential as barriers in preventing postoperative cardiac adhesion.

Unexpectedly, the overall observation showed inconsistent results after the animals were sacrificed and anatomized (Figures 7A–L, 9A–J). Large differences between individual animals were observed in the 0:100 group, i.e., some animals had no or slight adhesions, while the others were severe. However, mild adhesions were observed in both 30:70 and 50:50 groups. These results suggested that the 0:100 membrane was not suitable for the prevention of adhesions after cardiac surgery either.

Adhesion formation involves a series of complex pathophysiological reactions, such as the tissue damage, inflammatory responses, coagulation, fibrin deposition, fibroblast proliferation, collagen formation, and angiogenesis (Cannata et al., 2013; Kheilnezhad and Hadjizadeh, 2021), but the specific mechanism remains to be elucidated. Previous studies supposed that biomaterials could act as anti-adhesion barriers by blocking fibroblasts penetration (Jiang et al., 2014; Chen et al., 2015b), as the excessive proliferation of fibroblasts resulted in producing abundant collagen fibers to form dense and thick fibrous adhesions. However, in this study, histological staining showed that numerous inflammatory cells instead of fibroblasts infiltrated into the 0:100 membranes. It is known that the inflammatory response plays an important role in adhesion formation, e.g., inflammatory cell aggregation and inflammatory mediator release can promote fibroblast proliferation and thus aggravate adhesion (Pismensky et al., 2011; Hu et al., 2021; Zhang et al., 2021). Therefore, the inflammatory cells infiltration, not fibroblasts penetration, might be the real reason that PCL membranes failed to prevent postoperative cardiac adhesion.

Accumulation of acidic degradation products can elicit a severe inflammatory response. Polymers such as polyglycolic acid, polylactic acid (PLA), and their copolymers [poly (lactic-co-glycolic acid)] contain abundant carboxyl groups and can therefore release many acidic degradation products that trigger severe aseptic inflammatory responses (Lin et al., 2017; Shen et al., 2019; Tu et al., 2020). Unlike PLA, PCL has no carboxyl groups and therefore cannot induce an inflammatory response via the above mechanism. Further analysis showed that the pore size of materials might be the key factor to induce inflammatory responses (Madden et al., 2010; Chiu et al., 2011; Garg et al., 2013; Wang et al., 2014). Inflammatory cells are the smallest cells *in vivo*, the diameter was generally in the range of 6–20 μm, being largest for macrophages (14–20 µm) and smallest for lymphocytes (6–10 µm). And the 0:100 membrane had the largest pore size (9.9 ± 2.3 µm, Figure 1B) and was theoretically permeable to most inflammatory cells, whereas the pore sizes of 30:70 and 50:50 membranes were much smaller (4.8 ± 1.2 and 5.2 ± 1.4 µm, respectively, Figure 1B) than that of lymphocytes. In addition, the pore size of GT/PCL membranes will decrease further after implanting *in vivo* due to the hydrophilicity of GT (Figure 2), as the nanofibers can absorb large amounts of water and swell. Therefore, the small pore size enabled the 30:70 and 50:50 membranes not only block the invasion of fibroblasts (diameter = 20–30 µm), but also prevent the infiltration of inflammatory cells. Furthermore, compared to the 30:70 membrane, the 50:50 membrane might experience a larger pore size reduction due to its higher content of GT, which was strongly supported by the fact that some cells invaded into the 30:70 membrane, while no cells were observed in the 50:50 group 3 months after surgery (Figures 9L–Q).

Collectively, in consideration of good mechanical properties, excellent biocompatibility and effective anti-cell penetration ability, the GT/PCL 50:50 membrane is concluded the best one to prevent postoperative cardiac adhesion among all groups. As a physical barrier, it might have great potential in other biomedical applications, such as the prevention of non-cardiac postoperative adhesions, wound healing, and guided tissue or bone regeneration. Nevertheless, it still has some limitations. Though mild adhesions were observed between the GT/PCL 50:50 membrane and epicardium, there were still moderate or severe adhesions between the membrane and sternum, increasing the difficulties of reoperation as well. Loading medicine into the GT/PCL 50:50 membrane to further enhance its anti-adhesion efficacy will be investigated in our future study. In addition, the degradation rate of PCL is pretty slow which will increase the risk of infection and hinder tissue regeneration. Replacing or blending the PCL with easier absorbable materials such as polydioxanone (PDO) (Oh et al., 2011; Kwon et al., 2019) or Poly (glycerol sebacate) (PGS) (Vogt et al., 2019; Fu et al., 2020) might be more preferable.

## 5 Conclusion

Pure PCL (0:100) and GT/PCL (30:70, 50:50, 70:30) nanofibrous membranes were successfully fabricated via electrospinning. Through the assessments of physicochemical properties and biocompatibility, as well as evaluations of rabbit heart/liver/kidney functions, CRP/immunoglobulin levels and adhesion degree, we discovered that the 70:30 membrane failed to prevent postoperative cardiac adhesion because of its poor mechanical properties, and the pure PCL membrane could not effectively prevent either, because numerous inflammatory cells have infiltrated into the membrane which induced a serious inflammatory response. Only the GT:PCL 50:50 membrane exhibited excellent mechanical properties, good biocompatibility and effective anti-cell penetration ability, which might be a perfect barrier to prevent postoperative cardiac adhesion and show great potential in other biomedical applications such as the prevention of non-cardiac postoperative adhesions, wound healing, and guided tissue or bone regeneration. In addition, as a physical barrier, the ability to inhibit inflammatory cells infiltration, not fibroblasts penetration, might be the key consideration in design and fabrication of nanofibrous membrane to prevent postoperative adhesion.

## Data Availability

The raw data supporting the conclusion of this article will be made available by the authors, without undue reservation.
