# Erratum: Gelatin/Polycaprolactone Electrospun Nanofibrous Membranes: The Effect of Composition and Physicochemical Properties on Postoperative Cardiac Adhesion

**DOI:** 10.3389/fbioe.2022.862276

**Published:** 2022-02-11

**Authors:** Frontiers Production Office

**Affiliations:** Frontiers Media SA, Lausanne, Switzerland

**Keywords:** electrospinning, gelatin, polycaprolactone, postoperative adhesion, cardiac surgery

Due to a production error, the image used for Figure 8 was incorrect. Figure 6 was mistakenly duplicated and used in place of the correct image for Figure 8.

**FIGURE 8:**
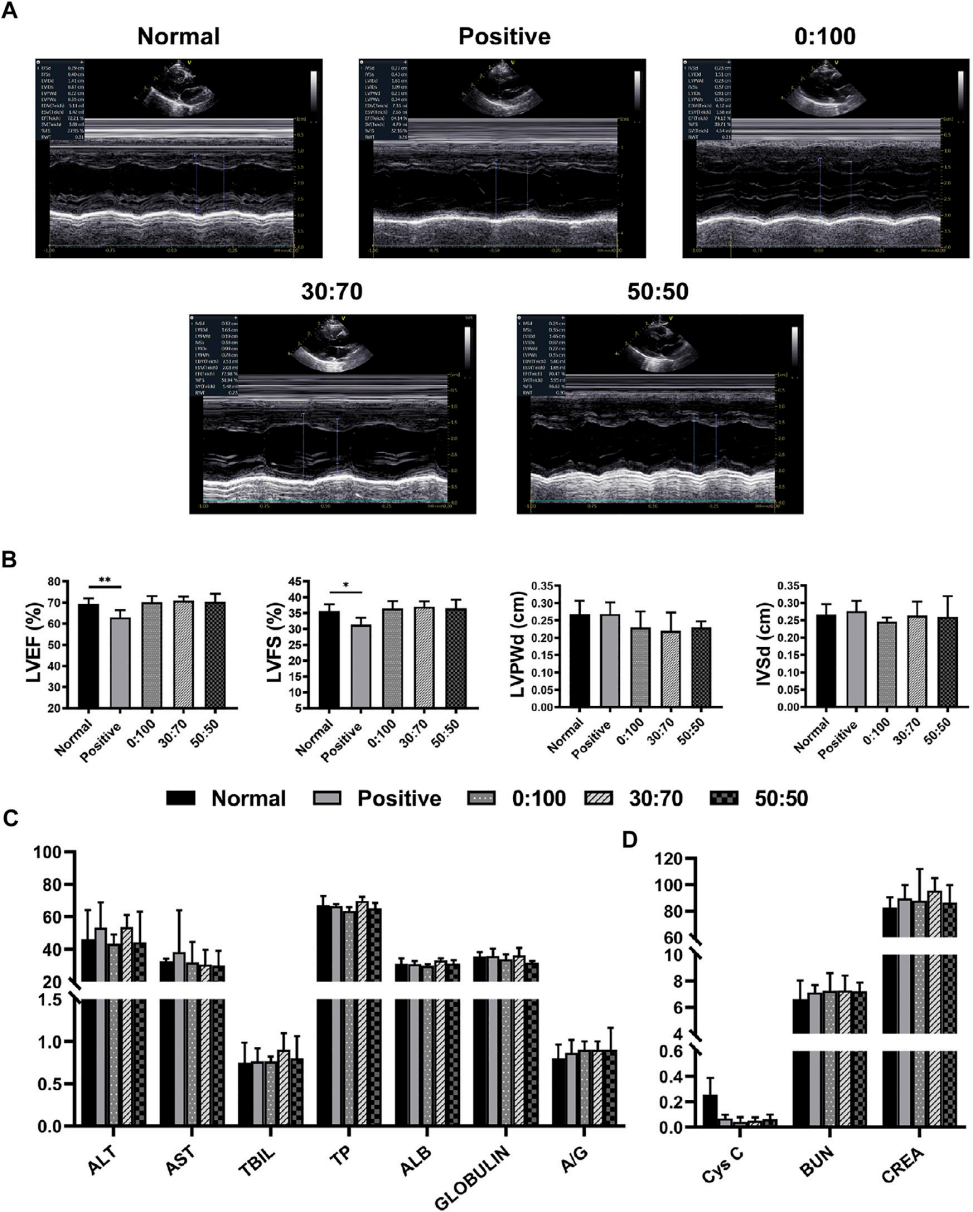
Three months after surgery **(A)** representative echocardiography images, **(B)** heart function and **(C,D)** liver/kidney function for normal and experimental groups. (*p < 0.05; **p < 0.01).

The correct Figure 8 appears below. The publisher apologizes for this mistake. The original version of this article has been updated.

